# Prevalence of sexually transmitted infections in female clinic attendees in Honiara, Solomon Islands

**DOI:** 10.1136/bmjopen-2014-007276

**Published:** 2015-04-28

**Authors:** M Marks, H Kako, R Butcher, B Lauri, E Puiahi, R Pitakaka, O Sokana, G Kilua, A Roth, A W Solomon, D C Mabey

**Affiliations:** 1 Clinical Research Department, Faculty of Infectious and Tropical Diseases, London School of Hygiene & Tropical Medicine, London, UK; 2 Hospital for Tropical Diseases, University College London Hospitals NHS Trust, London, UK; 3 Department of STI and HIV Prevention, Ministry of Health and Medical Services, Honiara, Solomon Islands; 4 National Referral Hospital, Honiara, Solomon Islands; 5 Eye Health Department, Ministry of Health and Medical Services, Honiara, Solomon Islands; 6 World Health Organization Country Office, Honiara, Solomon Islands; 7 Secretariat of the Pacific Community, Noumea, New Caledonia

**Keywords:** chlamydia, sexual health, gonorrhoea

## Abstract

**Objectives:**

This study sought to determine the prevalence of common bacterial sexually transmitted infections, including *Chlamydia trachomatis* and *Neisseria gonorrhoeae*, in women attending clinics in the Solomon Islands.

**Methods:**

We conducted a sexual health survey among women attending three nurse-led community outpatient clinics in August 2014, to establish the prevalence of bacterial sexually transmitted infections in female clinic attenders in Honiara, Solomon Islands. Vaginal swab samples were tested for infection with *C. trachomatis* and *N. gonorrhoeae* using a commercial strand displacement amplification assay. Serum samples were tested for syphilis.

**Results:**

We enrolled 296 women, aged 16–49, attending three clinics. Knowledge of safe sexual practices was high but reported condom usage was low. The prevalence of infection with *C. trachomatis* was 20%. The prevalence of infection with *N. gonorrhoeae* and syphilis were 5.1% and 4.1%, respectively.

**Conclusions:**

Bacterial sexually transmitted infections are a major health problem in the Solomon Islands. Interventions are urgently needed.

**Strengths and limitations of this study**

- We provide updated data on the prevalence of bacterial sexually transmitted infections (STIs) in a subset of the Solomon Islands population.
- In our sample of women attending clinics in Honiara, the prevalence of genital *Chlamydia trachomatis* infection was 20%. In participants aged less than 30 years, the prevalence was 28.2%.
- Co-infection with multiple bacterial STIs was found in 5% of participants.
- We did not test for non-bacterial STIs and are therefore likely to have underestimated the true burden of disease.
- Only women were enrolled in the study: the prevalence of STIs in males requires further study.

## Background

Sexually transmitted infections (STIs) are a major health problem in many countries, but in the developing world there are often limited data to inform public health strategies. *Chlamydia trachomatis* (CT) and *Neisseria gonorrhoeae* (GC) are the most common bacterial STIs worldwide.^1^ Infection with CT is frequently asymptomatic^2^ but is associated with a long-term risk of infertility.^3^ Bacterial STIs are also associated with an increased risk of HIV transmission.^4^ ^5^ The impact of comprehensive STI treatment strategies on HIV incidence has been mixed^6–8^ with some evidence to suggest that STI treatment strategies are most beneficial in settings with less established HIV epidemics.^9^

There are few data on the prevalence of STIs from the small Pacific Island states. STI surveillance studies conducted in antenatal clinics in six Pacific countries in 2004 demonstrated marked differences in prevalences of CT infection, ranging from 6.4% in the Solomon Islands to 29% in Fiji.^10^ The results of this survey prompted the formation of a regional STI Working Group, which has acknowledged the significant burden of disease and provided recommendations for enhanced control.^11^ An unpublished survey from 2008 showed a prevalence of genital CT of 16% in women attending antenatal clinics.^12^ A meta-analysis of studies undertaken in Papua New Guinea reported a community prevalence of 20–24% for CT, with higher prevalence reported in clinic-based studies.^13^ Studies in female sex workers in both Vanuatu and Papua New Guinea have reported prevalence rates for CT of over 30%.^13^ ^14^

The Solomon Islands is an archipelago of approximately 1000 islands, with a population of approximately 500 000,^15^ located between Papua New Guinea and Vanuatu. We conducted a sexual health survey of women attending general and antenatal outpatient clinics in Honiara, Solomon Islands.

## Methodology

Primary healthcare to the population of Honiara is provided by nurse-led community clinics that provide the full range of outpatient medical care, including general medical care, antenatal care and screening for STIs. Services are free at the point of care.

### Study population

A convenience sample of women aged 16–49 attending three public outpatient clinics in Honiara were invited to participate in the survey during a 10-day period in August 2014. Patients were recruited from attendees at both general and antenatal clinics. Nursing staff fluent in local dialects explained the study and obtained written informed consent. As the study was conducted alongside routine care in three busy outpatient clinics, we did not collect information on women who declined to participate.

For consenting women, study personnel completed an orally administered sexual health questionnaire, including information on age, marital status, occupation, number of lifetime partners and partners in the previous 12 months, previous STIs and condom use, and symptoms within the last month, and were asked to self-collect two vaginal swab samples. The first swab (intended for primary diagnostic purposes) was collected according to kit manufacturer's guidelines and transferred to the National Referral Hospital (NRH). The second swab was placed in a dry cryotube and stored at −80°C for future analysis. A blood sample was collected from all patients, from which serum was isolated at the NRH Medical Laboratory. In line with Solomon Islands national policy, HIV testing was restricted to patients attending within the context of an antenatal visit who had been offered voluntary testing and counselling.

### Laboratory testing

Diagnostic samples were tested within 24 h of collection. Serum samples were tested for syphilis at NRH in Honiara with a qualitative rapid plasma reagin (RPR) test for syphilis. Where the qualitative RPR was positive, a quantitative RPR and a *Treponema pallidum* haemagglutination (TPHA) test was performed. The combination of a positive TPHA and an RPR titre of ≥1/8 or more was considered diagnostic of syphilis. HIV testing was performed on blood samples from antenatal visit patients using the Alere Determine fourth generation combined antibody-antigen assay.

Vaginal swab samples were tested using the BD-Probetec strand displacement amplification assay for the simultaneous detection of CT and GC, according to manufacturer's guidelines. Positive and negative controls were included in each diagnostic run.

### Patient management

Patients were followed up and treated in line with Ministry of Health and Medical Services guidelines for the management of STIs.^16^

### Statistical analysis

A sample size of 246 was required to identify a prevalence of genital chlamydial infection of 20% with a precision of 5%. We report prevalence of STIs stratified by age (16–29 vs 30–49). Logistic regression was used to estimate unadjusted and adjusted ORs, controlling for confounders, for factors associated with infection with any bacterial STI. Age, level of education (primary, secondary or tertiary), area of residence (urban or rural), marital status, previous STI, number of partners and condom use in the past 12 months and the presence or absence of symptoms of an STI were considered to be variables of interest. Age and educational level were considered as a priori confounders when calculating adjusted ORs. For the purposes of analysis, patients reporting dyspareunia, abnormal vaginal discharge or a genital ulcer in the last month were considered to have symptoms consistent with an STI. All analyses were conducted using Stata V.13.1 (STATACorp).

## Results

296 women were enrolled in the study. The median age was 28 years (IQR 23–33 years) and 94% of participants were Melanesian. The median number of years of completed education was 8 (IQR 5–11). Further demographic data are presented in table 1.

**Table 1:** Demographic characteristics

	n	Per cent
Aged ≤29	175	61.6
Resident in urban area	273	92.9
Married/co-habiting	234	79
Educational level		
None	32	10.8
Primary	90	30.4
Secondary	140	47.3
Tertiary	34	21.6
Occupation		
Housewife	176	59.5
Paid employment	87	29.4
Unemployed	11	3.7
Question not answered	22	7.4
Nulliparous	64	21.6

### Sexual histories

The median age of reported sexual debut was 18 years (IQR 16–20). Seven per cent of participants reported sexual debut at an age of ≤14 years. The median number of lifetime partners was 2 (IQR 1–3) with 15% of individuals reporting 5 or more lifetime partners. Thirty-six women (12%) reported they had been unwilling parties in at least one episode of non-consensual sex. Only 26% of individuals reported ever having used a condom, and only 19% of individuals reported having used a condom in the past 12 months.

Twenty-seven women (9.1%) reported dyspareunia in the last month. Twenty-two women (7.4%) reported abnormal vaginal discharge and 18 women (6.1%) reported a genital ulcer in the last month, respectively. Thirteen women (4.4%) reported two or more symptoms in the last month. Twenty-eight women (9.5%) reported having been previously diagnosed with a STI of whom 11 (3.7%) reported having been treated for a STI in the past 12 months.

### Sexual health knowledge

In total, 273 (93%) women reported having heard of HIV. The commonest sources of information on HIV were the radio (57%), healthcare staff visiting villages (63%) and talks at local clinics (57%). Levels of knowledge about reducing the risk of HIV transmission were high; 245 (84%) and 249 (85%) women correctly identified that having a single faithful sexual partner and regular condom use, respectively, could reduce the risk of HIV transmission, while 264 (90%) women were aware of the risk of mother-to-child HIV transmission associated with pregnancy and breast feeding. In total, 213 (73%) women reported that they had access to confidential HIV testing. Difficulty accessing clinics for testing was the most commonly reported reason for being unable to obtain a confidential HIV test, with concerns about the confidentiality of the result being uncommon.

### STI test results

CT infection was diagnosed in 60 (20.3%) women and GC in 15 (5.1%). Serological evidence of current syphilis was found in 12 women (4.1%). The prevalence of CT, but not GC or syphilis seropositivity, varied significantly between age groups (table 2). Five per cent of women were diagnosed with two or more bacterial STIs. All antenatal clinic attendees participating in the study (n=134) underwent HIV testing following voluntary counselling and testing. There were no diagnoses of HIV.

**Table 2:** Bacterial sexually transmitted infection prevalence by age group

	Overall n=296	16–29 years n=175	30–49 years n=121	p Value
Chlamydia	60 (20.3%, 95% CI 15.8% to 25.3%)	49 (28.2%, 95% CI 21.5% to 35.3%)	11 (9.2%, 95% CI 4.6% to 15.7%)	<0.001
Gonorrhoea	15 (5.1%, 95% CI 2.9% to 8.2%)	11 (6.3%, 95% CI 3.2% to 11.0%)	4 (3.7%, 95% CI 0.9% to 8.2%)	0.333
Syphilis	12 (4.1%, 95% CI 2.1% to 7.0%)	9 (5.1%, 95% CI 2.4% to 9.5%)	3 (2.8%, 95% CI 0.05% to 7%)	0.337

There was no significant association between a positive diagnostic test result for an STI, educational level, marital status, reported condom use in the past 12 months, reported STI symptoms or history of previous STIs. There was a trend towards an increased risk in women with a higher number of sexual partners in the previous year, but this association was not statistically significant (table 3).

**Table 3:** Risk factors for proven bacterial STIs

	n	Unadjusted OR (95% CI)	p Value	Adjusted OR* (95% CI)	p Value
Age ≤29	175	2.79 (1.48 to 5.23)	**0.001**	3.01 (1.55 to 5.85)	**0.001**
Resident in urban area	273	0.52 (0.06 to 4.44)	0.545	0.66 (0.07 to 5.82)	0.705
Married/co-habiting	234	0.67 (0.34 to 1.32)	0.246	0.64 (0.31 to 1.32)	0.228
Educational level					
None	32	1	–	1	–
Primary	90	1.88 (0.65 to 5.46)	0.25	2.0 (0.66 to 5.82)	0.229
Secondary	140	1.89 (0.68 to 5.27)	0.25	1.4 (0.48 to 4.0)	0.550
Tertiary	34	1.16 (0.32 to 4.2)	0.83	0.93 (0.24 to 3.53)	0.910
Previous STI	28	0.84 (0.33 to 2.17)	0.725	0.91 (0.34 to 2.41)	0.847
Reported condom use in previous 12 months	56	1.25 (0.64 to 2.43)	0.520	1.38 (0.69 to 2.78)	0.365
Currently symptomatic	45	0.64 (0.28 to 1.45)	0.288	0.64 (0.26 to 1.56)	0.327
Number of partners in past 12 months					
1	257	1.26 (0.41 to 3.90)	0.692	1.36 (0.42 to 4.40)	0.609
2	7	1.6 (0.22 to 11.49)	0.640	2.52 (0.32 to 20.08)	0.382
≥3	5	2.67 (0.33 to 21.73)	0.360	3.29 (0.38 to 28.7)	0.282

Bold typeface indicates statistically significant results. *Adjusted for age and educational level. STI, sexually transmitted infection.

## Conclusions

We conducted an STI and self-reported sexual behaviour survey in Honiara, Solomon Islands. Overall, there was a high prevalence of STIs, and a particularly high prevalence of CT infection. Twenty-eight per cent of women aged 29 or under were found to be infected with CT, and 5% of women were infected with two or more bacterial STIs. The prevalence of CT in this study is high, similar to that seen in female sex workers in other countries of the region, such as Vanuatu and Papua New Guinea, where the prevalence was found to be 33% and 36%, respectively.^13^ ^14^ It is higher in our participants than the 11.1% reported in a recent antenatal clinic survey in Papua New Guinea.^17^

Previous published and unpublished studies conducted in 2004^10^ and 2008^12^ reported a prevalence of CT in women attending antenatal clinics in Honiara of 6.4% and 16%, respectively. In both of these studies, first catch urine samples were frozen and shipped at −20°C to Melbourne, where they were tested using the Roche Cobas Amplicor platform. Self-collected vaginal swabs have a higher yield than first catch urine samples for both CT and GC, regardless of the diagnostic platform used.^18^ It is possible that the higher prevalence of CT infection reported in this study represents a true increase in disease prevalence since the previous surveys were conducted, an increased sensitivity of the diagnostic strategy, or both.

We did not find an association between current symptoms or reported sexual behaviours and infection with CT or any other STI. Previous studies in the region have noted associations between infection with CT and lifetime number of partners, marital status and commercial sex work.^10^ Discussion of sexual behaviours is a culturally sensitive issue in the Solomon Islands. Although information was elicited by local staff, participants may have been reluctant to disclose some behaviours, such as multiple partners. This, combined with the relatively small sample size of our study may have limited our ability to detect such associations.

Of particular interest, the Solomon Islands is endemic for trachoma, a blinding eye disease caused by infection with ocular strains of CT*.*^19^ ^20^

At the time of this study, the Solomon Islands was about to embark on azithromycin mass drug administration (MDA) as part of a strategy for the elimination of trachoma.^21^

The impact of azithromycin MDA on genital CT and other STIs has not been previously studied. While azithromycin should be effective in treating prevalent genital CT infections, the transmission dynamics of ocular and genital CT are markedly different, making the impact of MDA difficult to predict. Although clinically significant macrolide resistance has not yet emerged in CT, there is a potential risk of inducing drug resistance in other STIs, notably *Mycoplasma genitalium.*^22^ Studies to assess the impact of azithromycin MDA on genital infections are warranted.

Reported knowledge of HIV and safe sexual practices was high, with clinic staff and Ministry of Health radio broadcasts the major sources of information accessed by women. Despite this, reported condom usage was low. Disconnects between sexual health knowledge and behaviour have been noted in other settings.^23^ Further work to explore the disparity between knowledge and behaviour in this population, and to reduce risk-taking behaviour, is warranted. Confidence in the ability to access confidential testing was high, with only 8 women (2.8%) reporting concerns about confidentiality. Instead, low access to testing was perceived as the major barrier to HIV testing. This suggests that an expansion of the current voluntary counselling and testing programme would be likely to result in an increased uptake in HIV testing. Other approaches to increasing HIV testing, such as household testing, could also be considered, though our data suggest that the population prevalence of HIV infection is low.

This study has a number of limitations. First, we report data only on women presenting to clinics in Honiara. The prevalence of infections and high-risk behaviours in the male population is unclear and not examined in the current study, but will influence the disease burden observed in women. In addition, it is likely that important factors such as the level of education, sexual behaviours and HIV risk vary between the predominantly urban population studied here and the rural population of much of the country. Second, we did not capture information on individuals who declined to participate in the study. It is possible that this population differs from those who agreed to participate and this may affect the generalisability of our findings. Third, the interpretation of syphilis serology is challenging in a country which is also endemic for yaws.^24^ Nine of the 12 women diagnosed with syphilis in this study had high RPR titres (1:32), however, making it less likely that this finding was due to childhood yaws. Finally, we tested only for bacterial STIs and did not test for common viral and protozoan STIs, such as herpes simplex virus, human papilloma virus and *Trichomonas vaginalis*. As a result, we are likely to have underestimated the total burden of STIs in this population.

In conclusion, we found a high prevalence of genital CT infection in women presenting to clinics in Honiara. The prevalence found in this study is markedly higher than in previous STI surveys in the country and is now among the highest in the region, equivalent to prevalences in high-risk groups in other countries. This very high CT prevalence suggests that further interventions are required to address the problem.
